# Understanding Aroma Release from Model Cheeses by a Statistical Multiblock Approach on Oral Processing

**DOI:** 10.1371/journal.pone.0093113

**Published:** 2014-04-01

**Authors:** Gilles Feron, Charfedinne Ayed, El Mostafa Qannari, Philippe Courcoux, Hélène Laboure, Elisabeth Guichard

**Affiliations:** 1 INRA (Institut National de la Recherche Agronomique), UMR1324 Centre des Sciences du Goût et de l'Alimentation, Dijon, France; 2 CNRS (Centre National de la Recherche Scientifique), UMR6265 Centre des Sciences du Goût et de l'Alimentation, Dijon, France; 3 Université de Bourgogne, UMR Centre des Sciences du Goût et de l'Alimentation, Dijon, France; 4 LUNAM University, ONIRIS (Ecole Nationale Veterinaire Agroalimentaire et de l'Alimentation), USC “Sensometrics and Chemometrics Laboratory”, Nantes, France; Duke University, United States of America

## Abstract

For human beings, the mouth is the first organ to perceive food and the different signalling events associated to food breakdown. These events are very complex and as such, their description necessitates combining different data sets. This study proposed an integrated approach to understand the relative contribution of main food oral processing events involved in aroma release during cheese consumption. *In vivo* aroma release was monitored on forty eight subjects who were asked to eat four different model cheeses varying in fat content and firmness and flavoured with ethyl propanoate and nonan-2-one. A multiblock partial least square regression was performed to explain aroma release from the different physiological data sets (masticatory behaviour, bolus rheology, saliva composition and flux, mouth coating and bolus moistening). This statistical approach was relevant to point out that aroma release was mostly explained by masticatory behaviour whatever the cheese and the aroma, with a specific influence of mean amplitude on aroma release after swallowing. Aroma release from the firmer cheeses was explained mainly by bolus rheology. The persistence of hydrophobic compounds in the breath was mainly explained by bolus spreadability, in close relation with bolus moistening. Resting saliva poorly contributed to the analysis whereas the composition of stimulated saliva was negatively correlated with aroma release and mostly for soft cheeses, when significant.

## Introduction

During eating, the first food transformations occur in the mouth and constitute preliminary steps of a series of reactions leading to digestion. Food breakdown occurring in the mouth not only facilitates food ingestion as a first step for digestion, but also contributes to the release of the stimuli responsible for the perception. However food breakdown not only depends on food structure but is also subject to inter-individual variations, which could explain the high differences observed on *in vivo* aroma release curves during food mastication.

So far, the relationships between physiology of mastication (including saliva) and aroma release have not been clearly established because the investigations have often been conducted on a limited number of subjects and on one specific oral physiological aspect.

In the literature, the role of oral mechanisms and processes in flavour release has mainly been described through the development of different mathematical models. The simplest cases were those applied to liquid samples not subjected to a mastication process, or to chewing gums. In addition to the simple release equations considering the effect of dilution with saliva [1] mathematical models for flavour release during drinking were developed based on the physiology of breathing and swallowing [2]. The first attempts to include mastication characteristics into the models were restricted to brittle foods that are fragmented during chewing and the simulation programme modelled chewing and swallowing as periodic events with characteristic frequencies [3], which are not comparable to mastication data for real subjects. In 2003, Wright *et al.* [4], and Wright and Hills [5] proposed a probabilistic model to describe the masticatory cycles with the aim of predicting the generation of in-mouth exchange area from the aroma release. They assumed that transfer of flavour from the saliva into the headspace was very fast compared to the transfer from the bolus into the saliva. However, this model considered neither the effect of breathing and swallowing nor the adhesion phenomena. More recently, a model of flavour release during the eating process was established, based on mass balance in each compartment (mouth, pharynx, nasal cavity, product in mouth or in pharynx) including ingoing and outgoing mass fluxes together with the swallowing events [6]. The model, which was validated using *in vivo* aroma release data showed that volatile compound concentration profiles in the nasal cavity are highly dependent on the breathing rate, the mouth volume, and the time of velopharyngeal closure between two swallows. This model was further improved to account for the residual amount of product coating the pharynx [7] allowing the validation of the influence of saliva dilution. All these approaches using mechanistic models need to be confronted with experimental data, which are not always available. More recently an extension of the model developed by Doyennelle *et al.* [7], was developed [8] by taking account of the mastication process during cheese consumption and the simulations issued from the model were compared to experimental data on ethyl propanoate (EP) release from cheeses previously obtained [9]. This mechanistic approach allowed highlighting that among the different parameters of the model, saliva incorporation into the bolus, duration of mastication and velopharynx opening had a major influence on the overall kinetics of aroma release. However, these parameters were not measured but calculated with the model and used as degree of freedom of the model for simulations. Moreover, the model was not able to fit the release curves obtained for a more hydrophobic aroma, nonan-2-one (NO). This was explained by a possible retention of this molecule by lubricated mucosa, which was not taken into account in the model.

By contrast to these deterministic approaches, an empirical modelling approach consists in performing statistical treatments on different data sets in order to explain flavour release. In this context, Partial Least Square (PLS) regression analysis is among the most used strategy of analysis to investigate the relationships among groups of variables. For instance this approach was successfully applied to explain fatty acid threshold perception in human by means of oral physiological characteristics [10]. PLS regression fits within the general framework of multiple linear regressions. It can also be seen as an extension of this framework to investigate the relationships between two blocks of variables: a block of response variables and a block of predictor variables. One of its main advantages is to efficiently deal with highly correlated variables even in situations where the number of variables largely exceeds the number of individuals. Obviously, this is a common occurrence in biomedical and biological studies. Another occurrence in this kind of studies is the presence of more than two blocks of variables. Very often, practitioners overlook this structure of the data and undergo separate PLS regression analyses on pairs of blocks. A more appropriate strategy of analysis is to undergo Multi Blocks-Partial Least Square (MB-PLS). The rationale behind this method of analysis is to generate latent variables (or components) from the response variables that are highly related to latent variables from the other blocks of predictive variables. More precisely, the latent variables in each block are computed as linear combinations of the variables of that block. MB-PLS aims at finding, step by step, underlying directions (i.e. components) in the response variables that are as much related as possible to components in the predictive block of variables [11]–[19]. Moreover, MB-PLS highlights the importance of each block in the prediction of the response variables. In the literature, MB-PLS approach has been described in some omics studies conducted with multitargeted approaches such as proteomic and metabolomics [20]. Up to our knowledge, in the field of food science, this statistical approach has not been widely disseminated within the scientific community though researches in the field lead to different data sets (sensory, physico-chemistry, physiology). However, recent studies use the PLS or the PLS –discriminant analysis (PLS-DA) to evaluate tomato liking [21], cheeses quality [22], or for linking gas chromatography – mass spectrometry (GC-MS) and proton transfer reaction – time of flight – mass spectrometry (PTR-TOF-MS) fingerprints of food samples [23].

In the present study, we propose to investigate, on well characterized subjects, the relation between aroma release during cheese consumption and different blocks of data corresponding to different characteristics of the oral processing events that can be involved in aroma release. Unlike the deterministic approach based on a mathematical analysis and modeling, we propose an empirical approach based on MB-PLS analysis. This approach will make it possible to prioritize the different blocks of variables involved in aroma release and to identify the most important variables within each block.

## Materials and Methods

The study protocol was submitted to an Ethics Committee and was approved on 17 April 2008 by the Comité de Protection des Personnes Est-1 (N°2008/15) and on 8 August 2008 by the Direction Générale de la Santé - France (N° DGS2008-0196).

A short summary of experimental plan is presented in Table 1.

**Table 1:** Overview of the experimental design of the study and the corresponding code for the cheese products.

Number of Subject	48 (25 men and 23 women)
Molecules (Ions) followed by APCI: abbreviation	Ethyl Propanoate (103): EP; Nonan-2-one (143): NO
Cheese product consumed	**lfS**: low fat, Soft; **lfF**: low fat, Firm;
	**hfS**: high fat, Soft; **hfF** : high fat, Firm

### Subjects

Forty eight subjects (23 females and 25 males aged between 22 and 60 years; average age: 40 years) participated to this study. Subjects were selected from a group of 100 volunteers based on their good dental and oral status (no missing teeth – except third molar, no occlusion disorder, no xerostomia, no medications that may impact saliva flow and composition), and on the repeatability of measured physiological parameters (salivary flow rate under resting and stimulated conditions, respiratory flux, salivary composition) [24] [25].

The subjects were not allowed to smoke, eat or drink starting one hour before the test session. All the subjects were informed of the observational nature of this study. They gave their signed consent and received a financial compensation for their participation to two sessions, each lasting about two hours.

Panel oral physiological variables are shown in Table 2.

**Table 2:** Oral physiological characteristics of the 48 subjects included in the study: Descriptive statistics.

Variables (unit)	Variables code in PLS projections	1^st^ Quartile	Median	3^rd^ Quartile	Mean	Standard deviation (n−1)
Salivary flux (ml/min.)	Sf_R	0.324	0.42	0.618	0.472	0.193
	Sf_S	1.79	2.37	3.4	2.57	1.06
Protein (mg/ml)	Prot_R	0.379	0.501	0.721	0.592	0.347
	Prot_S	0.865	1	1.21	1.04	0.31
Lipolysis (mU/ml)	Lipolysis_R	0.047	0.123	0.279	0.182	0.188
	Lipolysis_S	0.065	0.12	0.15	0.117	0.077
Amylase (U/ml)	Amylase_R	8.14	13	18.4	16.2	12.6
	Amylase_S	16.3	21.4	28.8	22.6	11
Lysozyme (U/ml)	Lysozyme_R	584	663	693	644	115
	Lysozyme_S	481	565	657	560	158
Proteolysis (U/ml)	Proteolysis_R	0.025	0.059	0.082	0.141	0.274
	Proteolysis_S	0.088	0.099	0.13	0.143	0.177
Sodium content (mM)	Na_R	2.08	2.88	4.08	3.31	1.99
	Na_S	7.61	11.7	18.5	14.5	9.8
Potassium Content (mM)	K_R	19.2	22.3	23.9	22.3	4.2
	K_S	15.2	16.7	19.1	17.4	3.15
Oral volume (cm^3^)	Oral_vol.	28.8	36.9	45.5	38.6	10.5

n = 48; R: resting, S: stimulated.

### Cheese products

Four processed model cheeses were designed with the following ingredients: cheddar, soft cheese, butter, melting salts, protein powder (casein), salt and water. Different textures were obtained by varying the water content (S = soft, F = firm) and varying the ratio of fat to dry matter from 25% for low fat cheeses (lfF and lfS) to 50% for high fat cheeses (hfF and hfS). The pH ranged from 5.27 to 5.55. The rheological properties of the cheeses were measured in a large deformation at a rotation of 0.01 rad.s^−1^ for 240 s using a Haake Viscotester (VT550 – Thermo electron GmbH, Karlsruhe, Germany). The breakdown stress (BS) corresponds to the maximum strength necessary to cause cheese breakdown, with the lowest values for the softest cheese (respectively 8129±469 and 8022±1309 Pa for lfS and hfS) and the highest values for the firmest cheese (respectively 15253±1231 and 15556±2307 Pa for lfF and hfF). The critical strain at breakdown (CSB) corresponds to the maximum rotation angle required to cause breakdown, with the lowest values for cheeses with the highest fat content (respectively 0.273±0.022 and 0.348±0.061 rad for hfS and hfF), and the highest values for cheeses with the lowest fat content (respectively 0.804±0.056 and 0.836±0.036 rad for lfS and lfF).

Model cheeses were flavoured with two aroma compounds differing in terms of their hydrophobicity (logP): nonan-2-one (NO: logP = 2.9) and ethyl propanoate (EP: logP = 1.4). They were added during cheese production at levels adjusted to achieve final concentrations in the cheeses around 6 mg.kg^−1^ for nonan-2-one and 25 mg.kg^−1^ for ethyl propanoate.

### *In vivo* aroma release measurement

*In vivo* aroma release was monitored as previously described [26] using Atmospheric Pressure Chemical Ionisation-Mass Spectrometry APCI-MS (ion trap Esquire-LC mass spectrometer, Bruker Daltonique, Wissembourg, France). Air was sampled from the nose at an average flow rate of 37 mL.min^−1^ *via* a fused silica capillary tubing (i.d. = 0.53 mm) heated at 150°C and to which a 5 kV positive ion corona pin discharge was applied. The two aroma compounds added to the model cheeses were monitored simultaneously according to their protonated molecular ion (MH^+^): ethyl propanoate (m/z = 103) and nonan-2-one (m/z = 143). Each subject was asked to position the plastic tube in one nostril (the same for all the experiments) and to breathe normally. This period (breath-blank phase) was used to record the potential residual signal of the previous sample until return to the baseline and to control the regularity of breathing. Subjects were instructed to place the piece of cheese ([2.25*2.25*1.1] cm; m = 6 g) in the mouth, and freely consume it while keeping the lips closed. The products were presented in a random order at 17°C. The temperature of the food bolus was measured before swallowing. The average temperature was of 35°C±2°C whatever the cheese and subject. All measurements were done in triplicate. Bread, apple and water were used as mouth cleansers between two tests.

**Figure 1:**
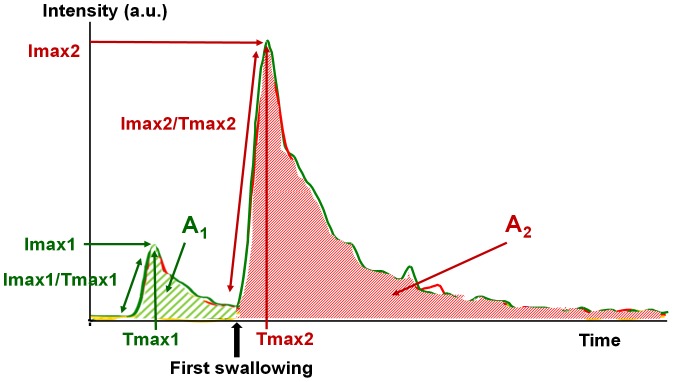
Typical aroma release curve profile. The release profile was separated in two release phases: before (phase 1) and after (phase 2) first swallowing. The quantity of aroma released (A1 & A2), the maximum intensity (Imax1 & Imax2), the time to reach maximum intensity (Tmax1 & Tmax2) and the release rate (Imax1/Tmax1 & Imax2/Tmax2) were extracted from the curve for each release phase.

After smoothing the curves to eliminate signal fluctuations due to the subjects' breathing patterns, two release phases were identified (Figure 1). The chewing phase (1) extended from placing the cheese in the mouth to the first swallowing, and the post-swallowing phase (2) extended from the first swallowing to the time at which the signal returned to its baseline level. For both release phases and for each aroma compound, three main parameters were extracted from each individual release curve: the area under the curve (A (a.u. : arbitrary unit)) representing the quantity of aroma released, the maximum intensity (Imax (a.u.)), the time to reach maximum intensity (Tmax (min)), the release rate (Imax/Tmax (a.u./min)) and the ratio of the quantity of aroma released between the two phases (A1/A2).

### Chewing activity

Chewing activity was monitored during cheese consumption, simultaneously to aroma release. The muscle activity of the superficial masseter and temporis muscles (left and right) during chewing was recorded by electromyography (EMG) using gold surface electrodes (Grass technologies, West Warwick, RI, U.S.A), at 382 Hz, then the signal was amplified and digitalized, as already described by Mioche *et al.* [27]. Number of chewing cycles (Nb_cycle), chewing duration (Chew_time expressed in s), mean amplitude of contraction (Ampl. Expressed in mV) and total muscle work (W_total expressed in mV.s^−1^) were calculated from EMG data. Mean amplitude of contration (Ampl.) corresponds to a mean calculated from the amplitude values of each chewing cycle registered in a whole chewing sequence [24].

### Bolus saliva content

The percentage of dry matter and water content were determined using an infrared dryer for all the cheeses and boluses obtained just before swallowing. For each subject and each cheese, the percentage of moistening (Moist_%) into the bolus was calculated as follows:
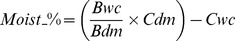
Bwc represents the bolus water content (%), Bdm represents the bolus dry matter (%), Cdm represents the cheese dry matter (%) and Cwc represents the cheese water content (%).

Three replicates per cheese and per subject were performed.

### Bolus rheology

Bolus rheological properties were measured as explained in more details in a previous paper [24]. Globally, the subjects were asked to chew the cheese samples until they were ready to swallow and to spit out the bolus into a truncated syringe. An aliquot of 3 mL of bolus at the syringe bottom was used for compression test to get a constant volume regardless of the cheese employed.

The compression device consisted of a mobile circle upper plate and a fixed circle lower plate. The test was performed with a compression rate of 1 mm.s^−1^. The fluid was subjected to a force F ranging between 0.01 N and 50 N. From the compression curve, particularly two phases were highlighted. A “flow phase” during which the suspension begins to flow and the particles move significantly in relation to one another at a height denoted as H_flow_(mm). Yield stress and viscous effects were described respectively by the parameters S_flow_ (Pa) and K_flow_ (Pa.s). A “particle phase” during which the mechanical response is governed by the particles the size of which is represented by a height denoted H_part_ (mm) and the yield stress component denoted S_part_ (Pa). At the end of the compression, H_end_ (mm) denotes the final height and S_end_ (mm^2^) the area generated under the maximal force. All measurements were done in triplicate.

### Mouth coating

Mouth coating (QRB_%), defined as the residual food that sticks to the oral surface after food ingestion, was quantified by the “mouth rinse” method [26]. The lipids of the residual food were quantified by the intensity of curcumin fluorescence in the rinse water.

The quantity of curcumin (Naturex, France), used as a food colour (E100), was added during cheese production to reach a level of 30 mg.kg^−1^ in the final cheeses. To measure the amount of cheese remaining in the mouth, each subject was asked to place a piece of cheese (6 g, at 17°C) in the mouth and to chew normally until they needed to swallow. The subjects swallowed without cleaning movement and then rinsed their mouth (with cleaning movements) with 4 mL of warm water at 50°C for 30 s, and spat it into a vial. This rinsing procedure was applied two times consecutively and the spittle was cumulated in the same vials. The fluorescence intensity of curcumin was quantified using a Perkin Elmer 1420 Multilabel Counter Victor 3 V at an excitation wavelength of 450 nm and an emission wavelength of 510 nm. All measurements were done in triplicate.

### Determination of maximal oral volume

An Eccovision acoustic pharyngometer (Hood Laboratories, USA) was used to measure the oral volume and was composed of four components: a wave tube, an electronic platform, a mouthpiece and a disposable filter. Reflectance pharyngometry was performed with a two-microphone imaging acoustic pharyngometer device, as described recently [28]. This device consists of two microphones and a horn driver mounted on a wave tube and connected to a PC-compatible computer with signal conversion capabilities. The signal was converted into the surface change (cm^2^) as a function of the length of the oral cavity (cm). The subjects held the mouthpiece in their mouth with their teeth against the flange and their tongue in a low position. To prevent air leaks, which could cause measurement errors, the subjects placed their lips over the flange, sealing the mouthpiece. The subjects were asked to breath with their nose during the measurement. Values are expressed in cm^3^ and correspond to the average of 10 measures.

### Collection of saliva samples and flux

The subjects were requested not to eat, drink or smoke starting from at least one hour before the collection of saliva samples.

Resting saliva was collected as previously described [28] by instructing the subjects to spit out the saliva every 30 seconds into a pre-weighed cup over a period of 5 minutes. For stimulated saliva, the subjects chewed a piece of Parafilm (0.5 g±0.2 g) for a period of 1 min and spit out the saliva every 30 s. The cups were weighted and the salivary flow rates were expressed in mL.min^−1^.

Immediately after collection, the saliva samples were standardized by a first step of centrifugation for 30 min at 15000-x g to remove bacteria and cellular debris and supernatants were then stored at −80°C to arrest metabolism until subjected to biochemical analyses [29].

### Biochemical analyses of saliva samples

#### Protein concentration

Protein concentration (Prot expressed in mg.ml^−1^) was obtained by standard Bradford protein assay Quick Start (Bio-Rad, France) using bovine serum albumin (Sigma-Aldrich, France) as standard for calibration.

#### Enzyme activities

All enzyme activities were expressed in International Enzyme Activity Units (U) per ml of saliva. One U is defined as the amount of enzyme that catalyses the conversion of 1 micromole of substrate per minute.

The lipolytic (Lipolysis), proteolytic (Proteolysis), lysozymal (Lysozyme) and amylolytic (Amylase) activities were determined as previously described and are detailed below [10], [30], [31].

Lipolytic activity was determined as followed. The buffer contained 50 mM Tris-HCl, pH 7.5, 4 mM CaCl_2_, 2 mM EDTA (ethylenediaminetetraacetic acid), 0.2% (w/v) NaTDC (sodium taurodeoxycholate), 1 mM PMSF (phenylmethylsulfonyl fluoride), 1 mM DTT (dithiothreitol) and 0.02% (w/v) sodium azide. The substrate solution was prepared by vortexing 19 volumes of the above buffer for 10 s with 1 volume of an ethanolic solution of 4-methylumbelliferyl 7-oleate (Sigma-Aldrich, France) for a final concentration of 1 mM. Reaction was carried out in microplate. Reaction started by adding 37.5 µl of saliva to 150 µl of substrate solution and 1.5 µl ethanol. A reaction of inhibition was also conducted for each sample by adding 1.5 µl of 125 µM ethanolic solution of THL (tetrahydrolipstatin) instead of ethanol. The intensity of fluorescence was followed continuously during 30 min at 37°C (excitation filter 355 nm, emission filter 460 nm) using a microtiter plate fluorometer (Victor 3-V, Perkin Elmer, France). The lipolytic activity was calculated from the difference between the average activity of slopes obtained for each sample without and with the lipase inhibitor THL. Activity was then read against a standard curve of umbelliferone. At each set of measurements a control of the linearity and proportionality of the reaction was also performed with commercial lipase (Aspergillus Niger Lipase, Fluka, France).

Proteolytic activity was determined using a Pierce Fluorescent Assay Kit (Pierce Biotechnology, Rockford, IL). A fluorescein labelled casein substrate liberates fluorescein fragment during proteolytic digestion which was followed during 60 min at 37°C (excitation at 494 nm/emission at 518 nm).

Lysozymal activity was determined using an EnzCheck Lysozyme Assay Kit (Molecular Probes, The Netherlands). The kit is based on the measure of the lysozyme activity on substrate Micrococcus lysodeikticus labelled with fluorescein. Intensity of fluorescence, proportional to lysozyme activity is read against a lysozyme standard and expressed in Unit/ml/min (excitation at 494 nm/emission at 518 nm).

Amylolytic activity was determined using CPNG3 Assay Kit (Biolabo, Maizy, France). The kit is based on the measure of the hydrolysis of 2-chloro-4-nitrophenyl malto trioside (CNPG3) into chloro-nitro-phenol (CNP), maltotriose and glucose. The rate of formation of CNP, directly proportional to the alpha-amylase activity, is measured at 405 nm against amylase standard.

#### Sodium and potassium analysis

The saliva samples were diluted to 1/20 (50 µL saliva in 950 µL filtered 18 mΩ Milli-Q-water (Millipore, Bedford, MA, USA)) and filtered through a membrane (pore size = 0.45 µm, C.I.L., Sainte-Foy-La-Grande, France).

The amounts of sodium (Na) and potassium (K) in saliva were determined by HPLC ionic chromatography using a Dionex ICS2500 ion chromatographic system (Dionex, Voisins le Bretonneux, France) as previously described [32] and expressed in mM. Quantifications were performed using calibration curves realised with sodium and potassium standard solutions ranging from 0.1 to 10 mM in 22 mM sulfuric acid (R^2^ = 0.999).

### Statistical analysis

Descriptive statistics on the four cheeses are reported in supplemental material and raw data are available on request.

#### Description and justification of the variables included into the MB-PLS analysis

The different variables used in the MB-PLS approach are presented in Table 3. They have been divided in six blocks. The Y block corresponds to the variables to be explained. It includes the most important variables related to aroma release measurement as explained above.

**Table 3:** Presentation of the different blocks of variables used in the MB-PLS analyses.

Block	Abbreviation	Definition of the variable
**Y**: aroma release parameters	A1	Area under the curve before 1^st^ swallowing
	A2	Area under the curve after 1^st^ swallowing
	A1/A2	Ratio between A1 and A2
	Imax1	Maximal intensity reached before 1^st^ swallowing
	Tmax1	Time to reach the maximal intensity before 1^st^ swallowing
	Imax1/Tmax1	Release rate before 1^st^ swallowing
	Imax2	Maximal intensity after 1^st^ swallowing
	Tmax2	Time of the maximal intensity after 1^st^ swallowing
	Imax2/Tmax2	Release rate after 1^st^ swallowing
**X1**: Bolus rheology	S_flow_	Yield stress at flow phase of compression curve
	S_part_	Yield stress at particle phase of compression curve
	H_part_	Bolus height at the beginning of the particle phase of compression curve
	K_flow_	Consistency at the flow phase, which reflects bolus consistency
	H_flow_	Bolus height at the beginning of the flow phase of compression curve
	H_end_	Bolus height at the end of compression
	S_end_	Area at the end of compression
**X2**: Coating- oral volume-% moistening	QRB_%	Quantity of product remaining in the oral cavity after swallowing in %
	Moist_%	Moistening of the products just before the swallowing in %
	Oral_vol.	Volume of the oral cavity
**X3**: Electromyography	Nb_cycle	Number of chewing cycle
	Chew_time	Chewing duration
	Ampl.	Mean amplitude of contraction
	W_total	Energy expended in chewing
**X4**:Resting saliva composition	Sf_R	Salivary flow at rest
	Prot_R	Amount of salivary proteins at rest
	Lipolysis_R	Amount of Lipolysis in saliva at rest
	Amylase_R	Quantity of Amylase in saliva at rest
	Lysozyme_R	Amount of Lysozyme in saliva at rest
	Proteolysis_R	Amount of Proteolysis in saliva at rest
	Na_R	Amount of sodium in saliva at rest
	K_R	Amount of potassium in saliva at rest
**X5**: Stimulated saliva composition	Sf_S	Salivary flow stimulated saliva
	Prot_S	Amount of salivary proteins stimulated saliva
	Lipolysis_S	Amount of Lipolysis in stimulated saliva
	Amylase_S	Quantity of Amylase in stimulated saliva
	Lysozyme_S	Amount of Lysozyme in stimulated saliva
	Proteolysis_S	Amount of Proteolysis in stimulated saliva
	Na_S	Amount of sodium in stimulated saliva
	K_S	Amount of potassium in stimulated saliva

**Y**: variables to be explained; **X**: explanatory variables.

The five other blocks correspond to the explaining variables (X1–X5).

X1 is related to bolus rheological parameters. These parameters have been highlighted in a previous paper as being the most relevant to discriminate the subjects on the basis of their masticatory behaviour [24]. Globally, these parameters reflect the consistency and the structure of the bolus at different stages of compressions with a final force (50 N) corresponding to that applied during pressure of the tongue to the soft palate at the swallowing time [33].

X2 is related to oral volume (Oral_vol), moistening of the product (Moist_%) and the remaining amount of product in the oral cavity after swallowing (QRB_%). These measures refer to the mouth coating and the generation of product exchange surfaces on the oral cavity mucosa after swallowing.

X3 corresponds to chewing behaviour parameters extracted from the EMG signals. The main variables previously identified as discriminating the most different panellists in terms of masticatory behaviour against cheese matrices were the total muscle work (W_total), the chewing duration (Chew_time), the number of masticatory cycle (Nb_cycle) and the mean amplitude of the contraction (Ampl.) [24].

The two last blocks (X4–X5) correspond to resting (R) and stimulated (S) saliva characteristics and composition. Whole saliva has been described as playing an active role on bolus formation, taste and aroma release [34]–[37] [38]. The choice to focus on both types of saliva is backed up by the different roles of both fluids related to food oral processing and food sensory perception. Resting saliva composition has been identified as involved in the prevention of oral dryness, lubrication of the oral mucosa, emulsion destabilisation [37], [39], fat perception [10], [31], [40] and bolus swallowing [41]. Stimulated saliva actively participates to the hydration of the bolus during chewing but also to the change of viscosity due to the action of some salivary enzymes such as alpha-amylase [42]. In term of salivary measured components, some refer more to the degradation of the product in the mouth (proteolysis, lipolysis, lysozyme and alpha-amylase), others to the interaction with aroma compounds (Na, K, proteins (Prot) and alpha-amylase) and still others to the oral moistening and clearance of the oral cavity (flow: Sf). A statistical test of comparison of means (Bonferroni-Dunn test; alpha = 0.05) was performed to spotlight differences between resting and stimulated saliva. Only proteolysis variable (Prot) turned out to show no significant difference between resting and stimulated saliva. Therefore, it was concluded that these two saliva samples were significantly different. Descriptive statistics of stimulated and resting saliva are reported in Table 2.

#### Statistical treatment

Statistical treatments of the present study were performed using the free software R 2.12.2 (Team 2011; http://cran.r-project.org/). The main R package used for multivariate data analyses was «pls 2.1-0» [43]. Statistical treatment consists to do an important pre-processing step as described by Hassani *et al.*, [44]. It consists briefly to: at first all the variables (belonging to both X and Y) are mean centered; then variables in X and Y are scaled block-wise to balance the sum of square contribution for different blocks; finally, in order to explore the systematic variation patterns in X which are likely to predict the systematic variation patterns in Y, PLS algorithm is applied. Then, the relationships between X blocks and Y and the importance of each block X for explaining Y were calculated as described elsewhere [45].

Median values across triplicates were used for statistical treatments. Missing data were replaced by the median value of the corresponding group. We chose the median values instead of the average values in order to avoid the influence of outliers and other unsuitable data [10].

In order to avoid cumbersome graphical displays, it was required for the correlation loadings plot that the correlations between the variables to be displayed and the retained components should be larger than a threshold value (R = 0.45); the remaining variables were ignored.

## Results

MB-PLS analyses were conducted on the different data sets to assess the extent to which the various blocks of variables explain the aroma release during cheese matrix consumption. The two aroma compounds nonan-2-one (NO) and ethyl propanoate (EP) were considered separately for statistical treatment.

### Choice and importance of the dimensions in the projection

For the choice of the number of components to be retained, a leave one out cross-validation procedure was performed. In this procedure, each sample is in turn set aside and a (MB-PLS) model was set up on the basis of the remaining samples. Thereafter, this model was used to predict the responses for the sample that was held out. Eventually, a statistic that assesses the differences (sum of squared) between predicted and observed responses was computed. Its evolution according to the number of components introduced in the model is indicative of the appropriate number of components to be retained since this statistic either reaches a plateau or starts to increase as the number of components increases. This indicates that the additional components fail to improve the model or, even worse, impede its performance because of the so-called over fitting problem. More details about cross validation can be found elsewhere [46]. A less formal way of assessing the relevance of the components is to consider the percentage of total variance in the Y block explained by the successive components. In our case study, the components beyond the third dimension explained less than 6%. Therefore, only the outputs of MB-PLS concerning the first three dimensions were explored. The block total variances (expressed as percentages) explained by MB-PLS components were used to assess the respective importance of the successive MP-PLS components. Two types of total variances were computed: those concerning the variables to be explained (Y block) and those concerning the explanatory variables (X blocks). The total variance of block Y was chosen to prioritize the importance of the three components (Table 4). In all the cases, the first MB-PLS component explains a larger variation than the two subsequent components. For ethyl propanoate (EP), the second component is more important than the third whereas for nonan-2-one (NO), the importance of dimensions 2 and 3 is more balanced. The firm cheeses have a larger explained variance on dimension 3 whereas soft cheeses have a larger explained variance on dimension 2. Moreover the total cumulated variance for the three dimensions is comprised between 34.82% and 53.25%. Considering the high variability and the importance of the error measurements in the data, this was believed to be large enough to draw tangible conclusions.

**Table 4:** Percentage of inertia on the first three dimensions.

Ethyl propanoate		Dim1	Dim2	Dim3	Nona-2-one		Dim1	Dim2	Dim3
lfS	**Y**	**20.57**	**13.27**	9.82	lfS	**Y**	**27.05**	**13.41**	7.97
	X	19.98	13.11	8.65		X	19.03	10.59	12.38
lfF	**Y**	**24.10**	**19.12**	8.98	lfF	**Y**	**27.47**	11.38	**14.40**
	X	15.85	10.88	13.78		X	16.20	14.77	9.35
hfS	**Y**	**21.02**	**14.32**	3.83	hfS	**Y**	**24.66**	**14.06**	5.91
	X	16.16	6.91	15.58		X	16.32	9.13	15.56
hfF	**Y**	**16.66**	**12.13**	6.03	hfF	**Y**	**23.17**	7.47	**9.01**
	X	19.38	8.58	10.48		X	20.00	13.11	6.33

lF: low fat, hF: high fat, S: soft, F: firm.

### Relative Importance of the blocks in the projection

**Figure 2:**
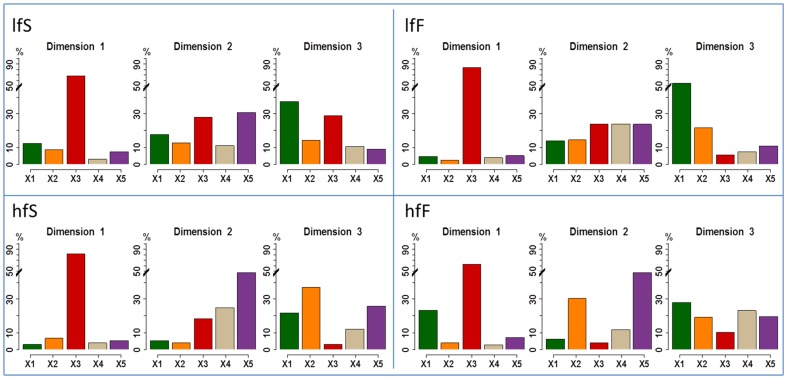
Bar charts representing the importance of the different blocks of variables (X1–X5) for the different dimensions obtained by means of MB-PLS analysis performed on ethyl propanoate release data set and for the four cheeses products. Green chart: rheology, Orange chart: coating, oral volume and % moistening, Red chart: EMG data, Grey chart: resting saliva composition, Violet chart: stimulated saliva composition.

**Figure 3:**
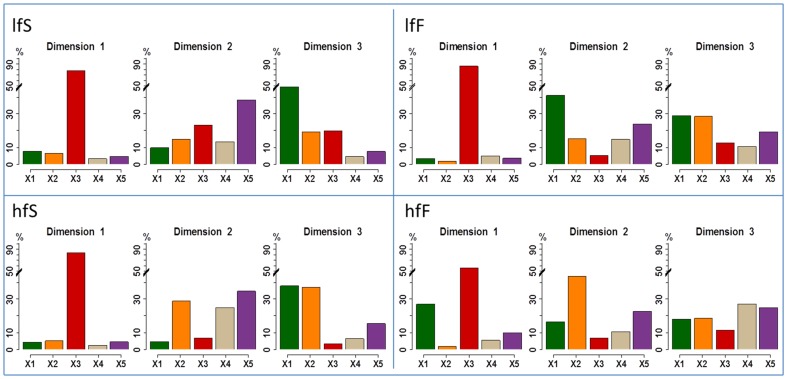
Bar charts representing the importance of the different blocks of variables (X1–X5) for the different dimensions obtained by means of MB-PLS analysis performed on nonan-2-one release data set and for the 4 cheeses products. Green chart: rheology, Orange chart: coating, oral volume and % moistening, Red chart: EMG data, Grey chart: resting saliva composition, Violet chart: stimulated saliva composition.

The importance of the blocks of variables for each MB-PLS dimension is shown in figures 2 and 3. For both molecules and whatever the cheese EMG block is always the most important block with an average percentage of variance explained comprised between 50% and 70% for the first dimension. For ethyl propanoate (EP) and whatever the cheese, stimulated saliva block is mainly reflected by the second dimension whereas rheology and coating-oral volume-moistening blocks are mainly reflected by the third dimension. For nonan-2-one (NO), the importance of the blocks on dimensions 2 and 3 is slightly different and depends on the cheese. For lfF cheese, rheology is mainly reflected by the second dimension whereas for hfF cheese it is the coating-oral volume-moistening block which loads on this dimension. The third dimension is mainly explained by the rheology block for lfS cheese and by the block of stimulated saliva for hfF cheese. For lfF and hfS cheeses the coating-oral volume-moistening block is almost as important as the rheology block.

Finally saliva at rest poorly contributes to the analysis when compared to stimulated saliva.

### Projections of the different variables

**Figure 4:**
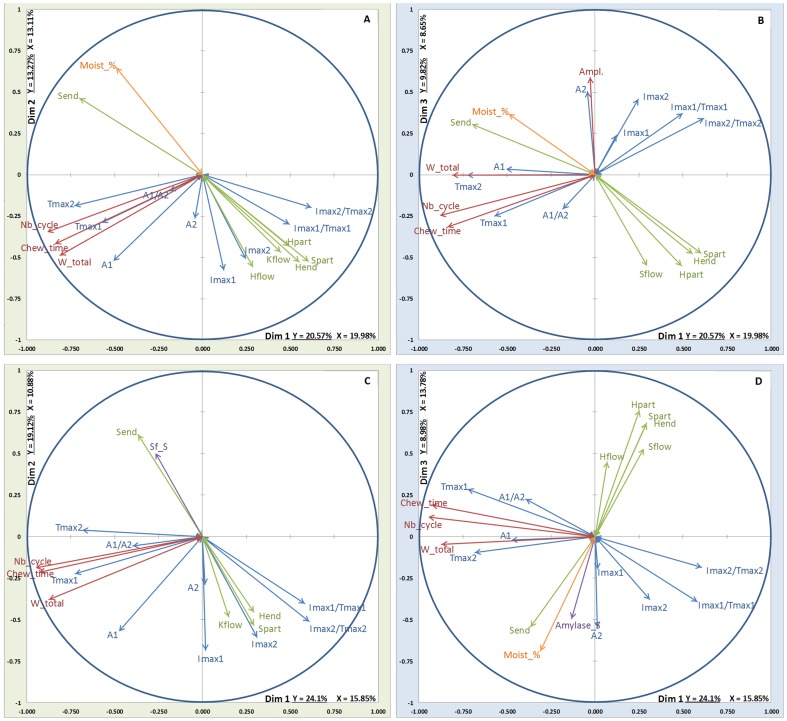
MB-PLS results on dim1/dim2 and dim1/dim3: relationships between the X-blocks of explanatory variables (Green arrows: rheology, Orange arrows: coating, oral volume and % moistening, Red arrows: EMG data, Violet arrows: stimulated saliva composition) and the Y-block of variables to be explained (Blue arrows: aroma release) for ethyl propanoate and low fat cheeses. Top: soft cheese; Bottom: firm cheese. A: EP_lfS (dim 1/dim2); B: EP_lfS (dim 1/dim3); C: EP_lfF (dim 1/dim2); D: EP_lfF (dim 1/dim3).

**Figure 5:**
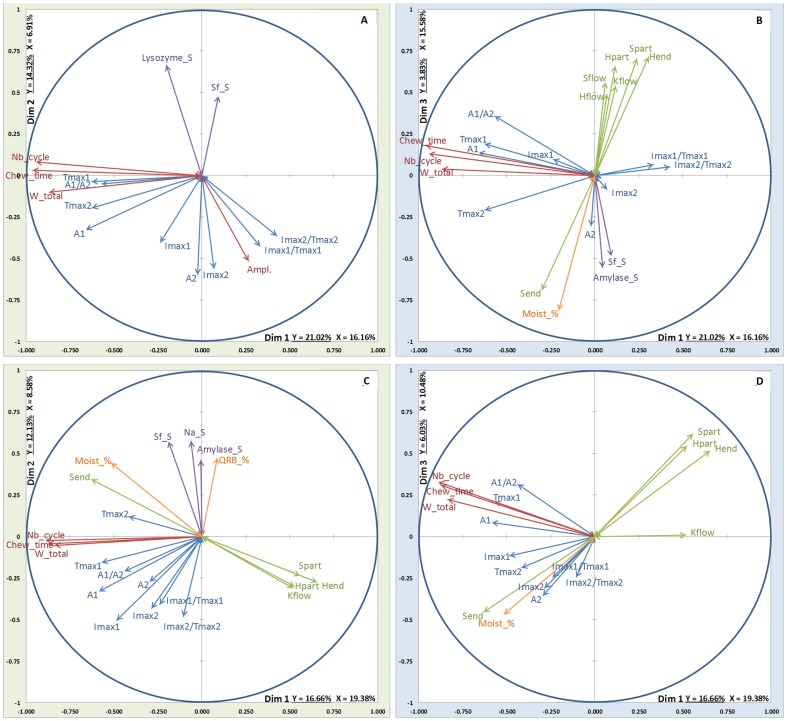
MB-PLS results on dim1/dim2 and dim1/dim3: relationships between the X-blocks of explanatory variables (Green arrows: rheology, Orange arrows: coating, oral volume and % moistening, Red arrows: EMG data, Violet arrows: stimulated saliva composition) and the Y-block of variables to be explained (Blue arrows: aroma release) for ethyl propanoate and high fat cheeses. Top: soft cheese; Bottom: firm cheese. A: EP_hfS (dim 1/dim2); B: EP_hfS (dim 1/dim3); C: EP_hfF (dim 1/dim2); D: EP_hfF (dim 1/dim3).

**Figure 6:**
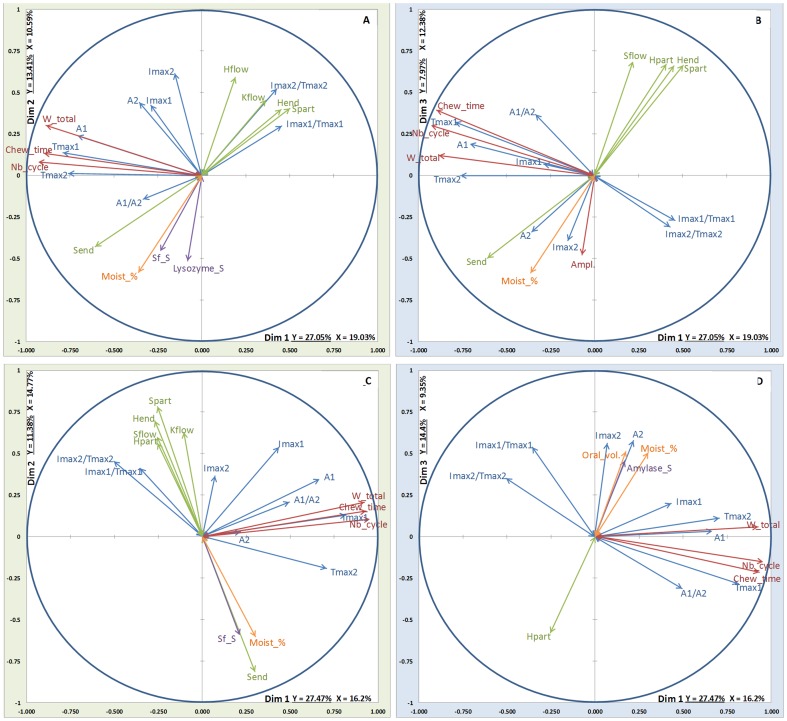
MB-PLS results on dim1/dim2 and dim1/dim3: relationships between the X-blocks of explanatory variables (Green arrows: rheology, Orange arrows: coating, oral volume and % moistening, Red arrows: EMG data, Violet arrows: stimulated saliva composition) and the Y-block of variables to be explained (Blue arrows: aroma release) for nonan-2-one and low fat cheeses. Top: soft cheese; Bottom: firm cheese. A: NO_lfS (dim 1/dim2); B: NO_lfS (dim 1/dim3); C: NO_lfF (dim 1/dim2); D: NO_lfF (dim 1/dim3).

**Figure 7:**
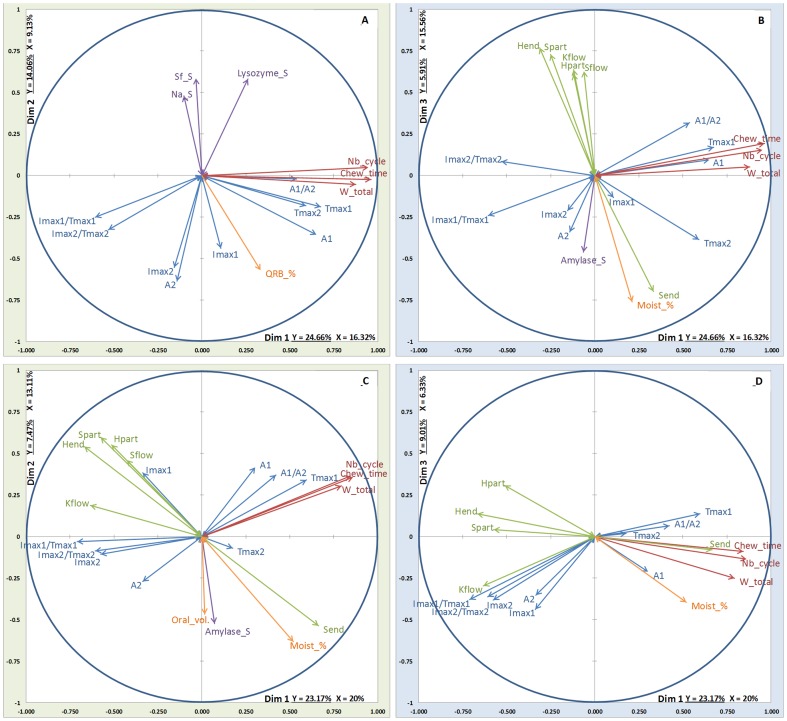
MB-PLS results on dim1/dim2 and dim1/dim3: relationships between the X-blocks of explanatory variables (Green arrows: rheology, Orange arrows: coating, oral volume and % moistening, Red arrows: EMG data, Violet arrows: stimulated saliva composition) and the Y-block of variables to be explained (Blue arrows: aroma release) for nonan-2-one and high fat cheeses. Top: soft cheese; Bottom: firm cheese. A: NO_hfS (dim 1/dim2); B: NO_hfS (dim 1/dim3); C: NO_hfF (dim 1/dim2); D: NO_hfF (dim 1/dim3).

The projections of the different variables from each block are presented in figures 4, 5, 6 and 7. On these figures, all the Y-variables are shown whereas, as stated above, only the explanatory variables with a correlation coefficient with one or the other of the components above 0.45 are depicted.

#### Electromyography block

Variables of electromyography block are depicted in red in the projection. For each projection, three explanatory variables (Nb_cycle, Chew_time and W_total) constituting the electromyography block are shown on the correlation plot, thus confirming the importance of this block. For aroma release variables (depicted in blue), Tmax1 and A1 are correlated with Nb_cycle variables Chew_time and W_total. The fourth variable (Ampl.) appears only in the EP_lfS (Fig. 4B), EP_hfS (Fig. 5A) and NO_lfS (Fig. 6B) projections and is not correlated with the three other variables. Aroma release variables Imax2 and Tmax2 seem to be better correlated with the variable Ampl.

#### Rheology block

Variables of rheology block are depicted in green in the projection. All the variables constituting the Rheology block seem to point to the same direction (indicating a positive correlation), except the variable S_end_ that points to the opposite direction than the other variables. The variables of this block (S_flow_, H_flow_, K_flow_, H_part_, H_end_, S_part_,) are correlated with the following aroma release variables: Imax1, Imax2, Imax1/Tmax1 and Imax2/Tmax2. This correlation is noticed for both molecules and especially for low fat cheeses (lfF & lfS) (Fig. 4 & 5).

#### Coating, oral volume and percentage of moistening block

This block is depicted in orange in the projection. For this block and nonan-2-one (NO), it can be noticed that QRB_% correlates on dimension 2 with the aroma release variables Imax2 and A2 for NO_hfS (Fig. 7A). Moreover, it is observed that the Moist_% variable is highly correlated with S_end_ (from the rheology block) whatever the projection, when they are depicted together.

#### Stimulated saliva block

For stimulated saliva block depicted in violet in the projection, the most frequently depicted variable is the salivary flow (Sf_S) and alpha-amylase (Amylase_S), except for EP_lfS (Fig. 4A&B) where the block is not depicted. Generally, these variables from saliva block are projected along the dimensions 2 or 3 and are more correlated with the percentage of moistening (Moist_%) and S_end_. This implies that the saliva block variables tend to be negatively correlated with the variables pertaining to aroma release. This is especially highlighted for EP_lfF (Fig. 4C), EP_hfS and EP_hfF (Fig. 5A, B & C) and NO_hfS (Fig. 7A).

## Discussion

The MB-PLS approach allowed us to relate the oral parameters to aroma release in a global statistical analysis. The objective of the present study was to test whether oral physiological characteristics, bolus rheology, saliva composition and mouth coating could explain the variability in aroma release observed in a significant number of human subjects. The statistical analysis prioritized the different blocks and highlighted the most relevant variables inside each block. This approach is likely to help setting up mechanistic hypotheses on aroma release.

### Masticatory behaviour

Among the four variables included in the masticatory behaviour block, three of them, namely, total work (W_total), chewing duration (Chew_time) and number of cycles (Nb_cycle) mainly explained the area under the curve during the chewing phase (A1) and the time to reach maximum intensity during this phase (Tmax1), whatever cheese and aroma compound. A lot of data in the literature are available evidencing that the number of chews, chewing work [47], [48], chewing frequency [49], longer chewing time and higher bursts number [50], [51] are positively correlated with high concentration of volatiles in the nose. The duration of mastication was found one of the parameters which had a major influence on the release dynamics obtained with a mechanistic model [8].

However our data highlighted that the average amplitude (Ampl.) was not correlated with other masticatory parameters. This parameter, when depicted on the projection, explained aroma release after swallowing for low fat cheeses and to a lower extend the rate of release. This was not evidenced in the literature yet. This finding could be explained by the fact that these types of cheeses are relatively firm (BS higher than 15000 Pa) and thus need a higher chewing force than high fat cheeses [50], [51]. This higher amplitude per burst might induce a quick release of aroma compounds, which explains the correlation with the rates of release. Moreover different chewing behaviours were already observed when eating confectionery chews [50], [51] and subjects displaying a higher chewing force («energic» eaters) presented a shorter chewing time which was responsible for a lower amount of aroma released in the nasal cavity. This was explained by a too short time to accumulate volatile in the oral headspace. In our study, the large average amplitude could also be associated to a higher bolus breakdown leading to higher bolus spreadability (S_end_) and lower particle size (H_part_), as shown on the projections EP_lfS (Fig. 4B) and NO_lfS (Fig. 6B), which explains the higher amount of aroma release during the post swallowing phase. It was also previously reported [24] that subjects who adapted their chewing behaviour to the product present a higher average amplitude (Ampl.) associated to a higher bolus spreadability (S_end_).

### Bolus rheology

Among the seven parameters included in the bolus rheology block, six characterized the bolus structure *i.e.*, representing bolus height (H_part_, h_flow_, H_end_), bolus consistency (K_flow_) and viscosity (S_flow_, S_part_). These parameters are highly correlated with food texture properties whatever the food [52], [53]. In our study, these six parameters are highly correlated with each other and mainly explain the release rate (Imax/tmax) and maximum intensity (Imax) of both aroma compounds from the firmer cheeses. These aroma compounds are released faster when the bolus is more consistent. The last variable, S_end_, is projected on the opposite side of the other variables. It represents the bolus area at the end of bolus compression, which is the bolus spreadability at the swallowing time, which is greater when bolus is less structured. S_end_ better explains the time to reach maximum intensity during the post swallowing phase (Tmax2) for the two aroma compounds and also the area under the curve during the post swallowing phase for nonan-2-one (NO), the most hydrophobic compound. This can be clearly explained by the higher affinity of nonan-2-one (NO) for fat, this compound being less released during the chewing phase [9], and thus more released during the post-swallowing phase, specifically in the case where a large quantity of the product remains in the mouth due to a higher bolus spreadability (S_end_). The higher release of this hydrophobic compound in relation with a higher bolus spreadability can be explained by a higher exchange area between food bolus and air, allowing more mass transfer to occur, which could induce a higher persistence of aroma in the breath [4].

### Bolus moistening and mouth coating

Bolus moistening (Moist_%) is depicted on the different plots suggesting an influence of this parameter on aroma release as mentioned also by Doyennette et al. [8]. A higher moistening induced a greater bolus spreadability (S_end_). These parameters being negatively correlated with the release rates (Imax/Tmax) and maximum intensities (Imax) of both aroma compounds from the low fat cheeses (lfS and lfF) and to some extend in relation with the higher amount of nonan-2-one (NO) released during the post swallowing phase (A2). Results obtained on the effect of bolus moistening cannot be simply explained by the Buttery equation [54]. Using this model, Doyennette *et al.* [55] were able to predict the changes in air/bolus partition coefficients for ethyl propanoate (EP) by incorporating increasing amounts of artificial saliva into model cheeses. They found a significant increase in EP air/bolus partition coefficient by increasing bolus moistening, whereas our results showed no effect of bolus moistening on area under the curve. Moreover, the same authors noticed a parabolic shape evolution of mass transfer coefficients by addition of saliva in the bolus issued from firm cheeses, with a minimum for the 50% dilution and a continuous increase for soft cheeses. Our results could not be explained by these predicted values, because the effect of bolus moistening on aroma release appears mainly dependent on the fat content and not on the firmness. Other factors than just dilution may explain *in vivo* aroma release. As a matter of fact, there should be a combined effect of saliva dilution and bolus spreadability on *in vivo* aroma release because these two parameters are always closely related. Using a mouth simulator Odake *et al.* [56] showed that dilution by saliva decreased the concentration of hydrophilic aroma compounds in the vapour phase. For more viscous samples such as dressings, which stick onto the inside cell wall, a higher aroma release was observed in comparison with simple emulsions, due to an increase in surface area.

Mouth coating (QRB_%) mainly explained the release of the more hydrophobic compound, nonan-2-one (NO) after swallowing for the high fat and soft cheese (hfS), the higher the coating, the higher the amount of this compound released after swallowing (A2), which can logically be explained by the fact that this hydrophobic compound is more soluble in fat than in water and thus is less released during the chewing phase from high fat cheeses. A higher amount of product remaining in the mouth will allow this compound to be released during a longer period of time (higher Tmax2) and thus lead to a higher total amount of aroma released (A2).

### Saliva flow and composition

Between the two saliva blocks, aroma release was only explained by stimulated saliva. Indeed, resting saliva is principally involved in the initial tasting of foods [57]. On food bolus structure, it has been described as being involved in destabilization and mouthfeel of liquid emulsions [37], [58], [59] [60] which does not involve a chewing behaviour. Stimulated saliva is particularly requested in bolus formation and structure during chewing of harder foods such as cheeses. Its incorporation into the bolus is essential for the coating of food particles to form a swallowable bolus [61]. Stimulated saliva block was projected essentially onto the second dimension for both soft and firm cheeses whatever the aroma compound and fat content. Salivary flow (Sf_S), alpha-amylase (Amylase_S), lysozyme (Lysozyme_S) and sodium (Na_S) were the most significant variables into the projection. In particular salivary flow (Sf_S) and the release rate (Imax/Tmax) and amount of aroma release (A1 & A2) are negatively correlated along dimension 2. In a time-intensity study conducted on 17 subjects, Guinard *et al.* [62], showed that the rate of release of flavour from cheery flavoured chewing gum can decrease when stimulated salivary flow increased. This effect was also noticed on *in vitro* studies conducted on beans, which showed a decrease of aroma release when volume of artificial saliva increased probably due to a dilution effect [63]. Moreover, stimulated saliva participates to oral clearance of remaining food [57], a higher flow leading to a higher removal of food debris in the oral cavity and thus to less aroma release. For other salivary variables, the effects are less significant. Lysozyme (Lysozyme_S) is only significant for soft cheese and is always depicted in the same direction as salivary flow (Sf_S) suggesting a similar role. The role of salivary lysozyme on food oral processing is not known for hard matrices. However, due to its positive charge, its effect in the structure of emulsion has been recently suggested [10]. For alpha-amylase (Amylase_S) and sodium content (Na_S), the effects are less significant and seem to vary depending on the cheeses and aroma compounds. These two parameters are projected in the same direction than the flow for ethyl propanoate (EP) and hfF (Fig. 5C) and for nonan-2-one (NO) and hfS (Fig. 7A), and in both cases in the opposite direction to the area under the curve after swallowing on the second dimension. These results confirm previous observations conducted in a mouth simulator which showed that the retention effect of salivary proteins (alpha-amylase and/or mucin) was higher than an eventual salting out effect of the salts [64].

## Conclusion

This study highlights the influence of some parameters which were not identified in previous ones.

Among the masticatory parameters which explain most of the differences in aroma release, the specific influence of mean amplitude on aroma release after swallowing was highlighted. This parameter was not evidenced in previous studies relating masticatory behaviour with aroma release.

Bolus rheology has a lower influence but it was noticed that the bolus spreadability explained the persistence of hydrophobic compounds in the breath, in close relation with bolus moistening. This aroma persistence was the subject of several studies involving biomechanical models. For example, the mechanistic model developed by Trelea *et al.* [6] explained the increased aroma persistence in the breath by an increase in the residual product layer thickness. However this model could not be validated by real data on residual product in the pharynx. This parameter was further introduced in a simplified biomechanical model to simulate the relative concentration of two aroma compounds in the nose of subjects consuming flavoured glucose syrups [7]. Even if the assumption stipulating that the post-deglutive pharyngeal residue diluted by saliva highly influences aroma release seems relevant, the bolus spreadability, which could be easily measured, should be taken into account in order to validate the model by experimental data. Finally, our work highlights the sole contribution of stimulated saliva on aroma release compared to resting saliva. This contribution is not only linked to a moistening of the matrix by water but also to other salivary components. However, one must bear in mind that saliva standardization procedure (centrifugation and congelation) as done in the current study may also affect some protein and/or enzyme levels and activities compared to whole saliva [29]. It is thus likely that such stimulated salivary components may contribute to aroma release from the food bolus differently in whole saliva. Moreover other salivary components not measured in the current study should be involved, as for instance, protein which is rich in prolin (PRP) that constitutes up to 75% of the proteins in stimulated saliva [65], [66].

MB-PLS approach made it possible to evidence the combined effect of bolus moistening and bolus consistency to explain the decreased rates of release of aroma compounds by saliva incorporation and the role of some saliva components. These results could not be explained by *in vitro* experiments and predictive models for aroma transfer. Our approach brings complementary results to those obtained using a mechanistic model with the same experimental data on the same cheeses [8]. This model fitted with 10 selected subjects and was successful only on one aroma compound, ethyl propanoate suggesting that the more hydrophobic aroma compound, nonan-2-one could be retained by lubricated mucosa. Moreover our statistical and integrated approach allowed the comparison of the effects observed for the two aroma compounds and a selection of the most important parameters to explain aroma release. Therefore, MB-PLS appears to be a powerful statistical approach in food science and aroma release in order to prioritize and identify novel important variables that should be taken into account in future scientific studies in the field.
